# Bifocal *β*-hCG-secreting CNS Germinoma in a 13-Year-Old Boy: Clinical–Biochemical Pubertal Discordance and Long-Term Outcome

**DOI:** 10.1155/crpe/5815092

**Published:** 2025-10-12

**Authors:** Corina Ramona Nicolescu, Sandrine Thouvenin Doulet, Lucie Bazus, Jean-Louis Stephan

**Affiliations:** ^1^Department of Pediatric Endocrinology and Diabetes, University Hospital Saint-Etienne, Avenue Albert Raimond, Saint-Priest en Jarez 42270, France; ^2^Department of Pediatric Hematology and Oncology, University Hospital Saint-Etienne, Avenue Albert Raimond, Saint-Priest en Jarez 42270, France

**Keywords:** gonadotrophin-independent puberty, human chorionic gonadotropin, intracranial germinoma, paraneoplastic syndrome

## Abstract

**Background:**

Intracranial germ cell tumors (GCTs) are rare in the pediatric population. They are classified as germinoma and nongerminomatous and may secrete specific proteins such as *β* subunit of human chorionic gonadotropin (β-hCG) and alpha-fetoprotein (AFP). When secreting *β*-hCG, they may induce gonadotropin-independent puberty (GIP), a clinical diagnostic clue that can precede neuroimaging findings.

**Case:**

A 13-year-old boy presented with a first generalized tonic–clonic seizure after six months of headaches, vomiting, polyuria, and polydipsia. Examination showed pubertal penile length with peripubertal testes. Laboratory assessment revealed panhypopituitarism with suppressed gonadotropins and elevated testosterone. Brain magnetic resonance imaging (MRI) demonstrated bifocal lesions (pineal and suprasellar) with obstructive hydrocephalus. Cerebrospinal fluid (CSF) sample obtained via temporary external ventricular drain (EVD) showed a normal cytology, confirming a *β*-hCG-secreting germinoma. The patient achieved remission after chemotherapy, neurosurgical resection, and cranial radiotherapy. He developed a posterior medulla relapse successfully salvaged with gemcitabine–paclitaxel–oxaliplatin, high-dose etoposide–thiotepa with autologous stem-cell transplantation, and craniospinal irradiation.

**Conclusion:**

Discordant clinical and biochemical puberty (penile enlargement with small testes and high testosterone with suppressed gonadotropins) should prompt *β*-hCG testing and targeted neuroimaging for suspected central nervous system GCTs. When raised intracranial pressure precludes lumbar puncture, CSF sampling via EVD is a safe alternative. Coordinated oncologic–endocrine care supports durable disease control with tailored long-term hormonal follow-up.

## 1. Introduction

Intracranial germ cell tumors (GCTs) are rare malignancies, accounting for 2%–3% of all primary central nervous system (CNS) tumors [1], predominantly affecting adolescents and young adults [2], with a male-to-female ratio of 2:1 [3]. They are commonly located in the midline region, may secrete *β* subunit of human chorionic gonadotropin (*β*-hCG), and are classified as germinomas or nongerminomatous GCTs [4].

A prolonged time to diagnosis is common, usually exceeding 6 months, although a recent study showed that delay in diagnosis does not adversely impact the outcome [5].

At presentation, the clinical features include endocrine dysfunction (most commonly, diabetes insipidus and pubertal disturbances), neurological symptoms (intracranial hypertension), and visual symptoms (Parinaud syndrome and visual field defects) [6].

Pubertal derangements vary by sex, age, and *β*-hCG secretion. Boys frequently show premature sexual development or discordant puberty [6, 7]. Girls predominantly present with hypogonadotropic hypogonadism [6], yet *β*-hCG-driven peripheral puberty has also been reported [8–12].

## 2. Case Presentation

A 13-year-old boy presented with a first generalized tonic–clonic seizure (5 min, loss of consciousness) following a 6-month history of progressive headaches, intermittent vomiting, polyuria, polydipsia, weight loss, and fatigue. He denied visual complaints. His past medical history was unremarkable. Family history was also noncontributory.

Clinical examination revealed an ill adolescent with a body weight of 29 kg (< 5^th^ percentile), height of 149 cm (10^th^ percentile), and body mass index (BMI) of 13.06 kg/m^2^ (< 3^rd^ percentile). His vital signs and systemic examination were otherwise normal. Genital examination showed an enlarged penis of 8 cm, bilateral testicular volume of 4 mL, and pubic hair without gynecomastia. The neurological examination was normal; funduscopic examination revealed bilateral papilledema.

Routine laboratory studies (full blood count, renal profile, liver function test, electrolytes, and serum calcium/phosphate) were within reference ranges (Table 1). Endocrine testing demonstrated complete anterior and posterior pituitary deficiency. Testosterone level was elevated, with suppressed gonadotropins (follicle-stimulating hormone [FSH] and luteinizing hormone [LH]) levels.

**Figure 1:**
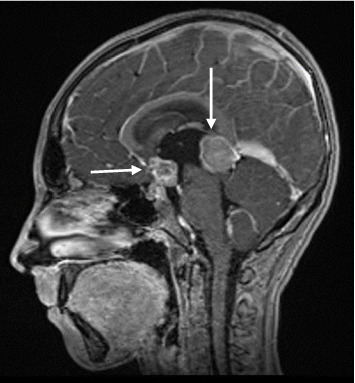
Sagittal T1 contrast-enhanced magnetic resonance images showing suprasellar and pineal lesions with heterogeneous enhancement. Moderate obstructive hydrocephalus was visible on additional views.

Brain magnetic resonance imaging (MRI) demonstrated two heterogeneous, contrast-enhancing lesions in the pineal (20 × 21 × 22 mm) and suprasellar (12 × 12 × 16 mm) regions, with moderate hydrocephalus (Figure 1). Spine MRI was normal.

Because papilledema and hydrocephalus contraindicated lumbar puncture, a temporary external ventricular drain (EVD) was placed and corticosteroids initiated, enabling safe cerebrospinal fluid (CSF) sampling. CSF cytology was negative for malignant cells, but *β*-hCG was elevated (Table 1). Serum *β*-hCG was 130 IU/L, and AFP was 4.3 ng/mL. A diagnosis of bifocal *β*-hCG-secreting CNS germinoma with secondary panhypopituitarism and raised intracranial pressure was established. Treatment approach aligned with contemporary consensus recommendations [4]: multimodal therapy comprising four cycles of ifosfamide, etoposide, and cisplatin, followed by complete neurosurgical resection and cranial radiotherapy (54 Gy). Early follow-up showed undetectable serum *β*-hCG and no residual disease (Table 1).

Hormone replacement therapy with hydrocortisone, desmopressin, and levothyroxine was progressively initiated within the first week after diagnosis. Growth hormone treatment was started 1 year after remission.

During surveillance, the patient experienced an intracranial relapse, as a contrast-enhancing lesion on the posterior aspect of the medulla oblongata. He was reinduced into remission with three cycles of gemcitabine, paclitaxel, and oxaliplatin, followed by high-dose etoposide and thiotepa with autologous hematopoietic stem-cell transplantation. Craniospinal irradiation was then delivered with dosimetric adaptation to previously irradiated proton fields (30 Gy craniospinal, 23 Gy cranial boost).

At 4 years of follow-up, MRI surveillance every 3 months has shown no recurrence.

Lifelong pituitary deficits were managed with growth hormone, hydrocortisone, levothyroxine, desmopressin, and testosterone.

At the time of writing, the patient is 18 years and 6 months old, with the final height of 162.7 cm (< 5^th^ percentile), and Tanner stage IV, with the testicular volume of 12 mL bilaterally. Final height was likely constrained by spinal proton irradiation and bilateral epiphysiodesis for severe genu valgum performed at the age of 16.

Key clinical lesson: The combination of neurological symptoms, pubertal dissociation (pubertal penile length with peripubertal testes), and panhypopituitarism with isolated high testosterone level was consistent with *β*-hCG-driven GIP due to CNS germinoma and should prompt targeted biochemical testing and neuroimaging.

## 3. Discussion

This case highlights a diagnostically valuable pattern in *β*-hCG-secreting CNS GCTs: discordant puberty features in male adolescents (pubertal penile length and peripubertal testicular volume) with panhypopituitarism and elevated testosterone level despite suppressed gonadotropins.

*β*-hCG germinoma secretion can precipitate premature sexual maturation, classically isosexual precocity in boys < 9 years and discordant puberty in older boys despite prepubertal gonadotropin levels. Two nonexclusive mechanisms are proposed, depending on two main tumor characteristics: location and the potential to produce *β*-hCG [13]. The anatomic pathway refers to the tumor location that may trigger early hypothalamic–pituitary activation (gonadotropin-dependent pubertal precocity) [14]. The biochemical pathway involves tumor-derived *β*-hCG, whose *β* subunit is homologous to LH and activates testicular LH receptors on Leydig cells, increasing testosterone synthesis independently of gonadotropin-releasing hormone (GnRH). In boys, GIP typically presents as a discrepancy between prepubertal testicular volume and pubertal penis length, prepubertal gonadotropins, and pubertal sex steroid levels.

As tumor detectability and *β*-hCG levels may fluctuate, GIP is considered a prodromal signal for GCTs; repeated hormonal testing and interval neuroimaging are warranted [15–17].

In girls, hypogonadotropic hypogonadism is common with suprasellar tumor location and hypothalamic–pituitary axis involvement, and *β*-hCG-mediated GIP is rare and physiologically complex. Three different biological hypotheses are discussed: (1) *β*-hCG could possess dual LH- and FSH-like activity, (2) tumor cells could produce a different substance with FSH-like activity, and (3) tumor cells might secrete *β*-hCG and simultaneously have aromatase activity, converting ovarian androgens to estrogens, thus inducing precocious pubertal development [10, 12]. Recently, a case report of suprasellar choriocarcinoma in a 7.5-year-old girl demonstrated the production of aromatase by tumor cells in immunohistological studies [8].

Peripheral *β*-hCG-producing tumors (choriocarcinoma of the liver, hepatoblastoma, and GCTs of the mediastinum) can similarly induce GIP in boys. In male adults with *β*-hCG-secreting tumors, gynecomastia was reported and might be secondary to the conversion of testosterone to estradiol via tumor cells aromatase synthesis [18–20]. Ovarian dysgerminoma with aromatase activity (and intratumoral estrogen synthesis) has been implicated in female precocious puberty [21–24].

Table 2 summarizes the characteristics of pediatric patients (boys and girls) with *β*-hCG-secreting intracranial GCTs (case reports compiled from PubMed, key words: *β*-hCG-producing tumor and early pubertal development).

In boys with intracranial *β*-hCG-producing tumors and sexual precocity, common findings included variable systemic symptoms/signs, a consistent pubertal discrepancy (small testes with penile enlargement), and similar biological profiles (suppressed and nonresponsive gonadotropin secretion and cerebral *β*-hCG secretion). Female GIP is an extremely rare event, and the relationship between precocious sexuality and CNS GCT is more complex and less explored.

## 4. Conclusion

We described an adolescent boy with bifocal *β*-hCG-secreting germinoma and overlapping symptoms, including neurological (pineal lesion) and endocrine (suprasellar lesion) dysfunction.

Discordant clinical and biochemical puberty (penile growth with small testes and high testosterone with suppressed gonadotropins) is a practical red flag for *β*-hCG-driven GIP in suspected CNS GCTs. Safe CSF sampling via EVD is appropriate when lumbar puncture is contraindicated by raised intracranial pressure.

Coordinated oncologic–endocrine care with structured MRI surveillance supports durable remission while managing endocrine sequelae.

## Figures and Tables

**Table 1:** Patient's biological characteristics and their evolution.

Laboratory test	Normal range	At presentation	6 months later	12 months later
TSH	0.50–4.30 mIU/L	2		
T4	12.6–21 pmol/L	6.7		16.5
IGF1	90–589 μg/L	68		73
IGFBP3	3.1–9.5 mg/L	1.51		
Cortisol	74–507 nmol/L	36		407
ACTH	10–60 pg/mL	11		
DHEAS	570–4100 ng/L	524		
LH	IU/L	< 0.3	< 0.3	< 0.3
FSH	IU/L	< 0.3	< 0.3	< 0.3
Inhibin B	74–470 pg/mL	24		
Testosterone	10.4–26 nmol/L	33	0.07	< 0.13
Prolactin	86–324 mIU/L	3710		910
Urinary osmolality	40–1400 mOsm/kg	153		411

*Tumor markers*				
AFP	ng/mL	4.3 (serum)		< 10
	< 10	0.8 (CSF)		
β-hCG	IU/L			
CSF	< 0.6	12	< 0.6	< 0.6
Serum	< 2	130		

Abbreviations: ACTH, adrenocorticotrophic hormone; AFP, α-fetoprotein; CSF, cerebrospinal fluid; DHEAS, dehydroepiandrosterone sulfate; FSH, follicle-stimulating hormone; β-hCG, β-human chorionic gonadotropin; IGF1, insulin-like growth factor 1; IGFBP3, insulin-like growth factor binding protein 3; LH, luteinizing hormone; T4, thyroxine; TSH, thyroid-stimulating hormone.

**Table 2:** Summary of reported cases of intracranial *β*-hCG-producing tumor and early pubertal development.

	Age	Clinical/pubertal features	Biological features	Diagnosis	Reference
*Boys*					
Case 1	5.8 years	Phallic growth, facial hair (tanner stage II/III), accelerated growth velocity	LH < 0.1 IU/L, FSH < 0.1 IU/L, testosterone 4.48 ng/mL, serum *β*-hCG 7.2 mIU/mL, CSF *β*-hCG 15.6 IU/L	Pineal *β*-hCG-secreting tumor (teratoma)	[25]
Case 2	5 years	Penile enlargement, pubic hair (tanner stage III), rapid linear growth	LH < 0.2 mIU/mL, FSH < 0.2 mIU/mL, testosterone > 1500 ng/dL, 17-hydroxyprogesterone 633 ng/dL, serum *β*-hCG 695 mIU/mL, CSF *β*-hCG 851 IU/L	Pineal *β*-hCG-secreting GCT	[26]
Case 3	6.2 years	Penile enlargement (7.5 cm), pubic and axillary hair (tanner stage III), growth acceleration, headaches	LH < 0.2 IU/L, FSH 0.1 IU/L, testosterone 6.85 ng/mL, serum *β*-hCG 15 IU/L, CSF *β*-hCG 4.8 IU/L	Pineal GCT (95% mature teratomatous component and < 5% germinoma component)	[27]
Case 4	5.7 years	Phallic enlargement, pubic hair, growth acceleration	LH < 0.2 IU/L, FSH 0.4 IU/L, testosterone 7.1 nmol/L, CSF/serum *β*-hCG ratio > 1.6	Nonsecreting GCT 8½ years after GIP diagnosis	[10]
Case 5	9.5 years	Fully grown pubic and axillary hair, right hemiparesis		Capsuloganglionic germinoma	[28]
Case 6 (15 boys)	10.8 years (mean age)	Sexual precocity (12 boys), growth retardation, polyuria and polydipsia, headache and vomiting, abnormal gait and hemiplegia, visual deficits	Suppressed gonadotropin response to GnRH stimulation, testosterone levels 6.7–63.8 nmol/L, serum *β*-hCG 120 IU/L	Tumors: germinoma (47%), mixed type (42%), immature teratoma (2%), choriocarcinoma (2%), location: hypothalamic–pituitary region, hypothalamus-pineal, pineal, hypothalamus -ventricular wall-pineal, basal ganglia, thalamus, cerebellar vermis	[29]
Case 7	9.5 years	Enlargement of penis (8 cm), testes volume 5 mL, pubic hair (tanner stage IV)	Testosterone 22 nmol/L, LH 0.128 IU/L, serum *β*-hCG 22.47 IU/L, CSF *β*-hCG 47.9 IU/L	Bilateral basal ganglia germinoma	[30]
Case 8	10 years	Increase in penis size, premature pubarche, facial acne	Testosterone 17.54 nmol/L, LH 0.1 IU/mL, FSH 1.0 IU/mL, serum *β*-hCG 431 mU/mL, CSF *β*-hCG 1609 mU/mL	Suprasellar germinoma	[31]
Case 9	7.5 years	Enlargement of penis, appearance of pubic hair, increasing growth rate, increased aggressiveness	Testosterone 54 nmol/L, suppressed LH and FSH peaks during LHRH test, serum *β*-hCG 115 IU/L, CSF *β*-hCG 814 IU/L	Pineal immature teratoma and choriocarcinoma	[32]
Case 10	7 years	Enlargement of penis, pubic hair (tanner stage II), linear growth acceleration	Testosterone 13.8 nmol/L, blunted, prepubertal response on LHRH test, CSF *β*-hCG 174 IU/L, serum *β*-hCG 8 IU/L	Pineal malignant teratoma	[32]
Case 11	9.7 years	Short stature (9.7 years), additional hormonal deficits (12 years), neurologic symptoms (13.6 years)	Testosterone 7.6–36 nmol/L, LH < 0.5 IU/L, FSH < 0.5 IU/L, serum *β*-hCG 3800 IU/L, CSF *β*-hCG 38 IU/L	Suprasellar choriocarcinoma	[33]
Case 12	8 years	Tanner stage III, polyuria, left abducens nerve palsy	Testosterone 0.27 nmol/L, LH 76.16 IU/L, FSH 4.87 IU/L, serum *β*-hCG 268 IU/L	Intrasellar mixed GCT	[34]
Case 13	5.3 years	Testicular and penile enlargement, pubic hair (tanner stage II), pain in the right thigh	Testosterone 0.45–0.83 nmol/L, FSH, LH not responsive to LH-RH test, serum *β*-hCG 38-92.9 lU/L, CSF *β*-hCG 12630 lU/L	Spinal cord germinoma	[35]
Case 14	10 years	Rapid pubertal development, peripubertal testicular volume, polyuria, decreased growth rate	Testosterone 0.9 nmol/L, anterior pituitary deficiency, serum *β*-hCG 24.5 IU/L	Suprasellar germinoma	[36]
Case 15	10.6 years	Genitalia stage III, pubic hair stage IV, testes 3 mL bilaterally, headache, vomiting, parinaud syndrome	Testosterone 28.4 nmol/L, serum *β*-hCG 1750 IU/L	Pineal germinoma	[37]
Case 16	9 years	Enlargement of phallus (10 cm), small testes, headaches, vomiting, blurred vision	Testosterone > 69 nmol/L, LH > 70 IU/mL (cross reactivity with *β*-hCG), FSH < 0.8 UI/L, Β-hCG 4825 IU/L	Pineal *β*-hCG-secreting tumor	[38]

*Girls*					
Case 1	7.5 years	Breast enlargement, tanner stage III, visual field disturbances	Undetectable LH, FSH, estradiol; pan-hypopituitarism; serum *β*-hCG 48,800 IU/L, CSF *β*-hCG 28,200 IU/L, normal AFP levels	Suprasellar choriocarcinoma	[8]
Case 2	5 years	Breast and pubic hair development	GnRH-independent precocious puberty, hyperprolactinemia, hypothyroidism, mild testosteronemia	Suprasellar GCT	[9]
Case 3	8 years	Left breast tenderness	LH < 0.2 IU/L, FSH < 0.5 IU/L, no response to GnRH stimulation, estradiol 228 pmol/L, androstenedione 3.1 nmol/L, serum *β*-hCG 187 IU/L, CSF *β*-hCG 81 IU/L	Germinoma in the suprasellar cistern, extending into the anterior sella turcica	[10]
Case 4	6 years 3 months	Tanner stage II, polyuria, polydipsia, headache, vomiting	LH < 2 IU/L, FSH < 2 IU/L, no response to LHRH stimulation, estradiol 31–47 ng/mL (< 10), serum *β*-hCG 1200 IU/L	hCG-secreting suprasellar immature teratoma	[11]
Case 5	5 years	Bilateral breast enlargement. Headache, nausea, vomiting	Low estradiol levels, LH 306.2 mIU/L, FSH 8.9 mIU/L, negative LHRH stimulation test, CSF *β*-hCG 200 ng/mL	Ectopic pinealoma of two-cell pattern	[12]

*Note:* CSF, cerebrospinal fluid.

Abbreviations: *β*-hCG, *β*-human chorionic gonadotropin; FSH, follicle-stimulating hormone; GCT, germ cell tumor; GnRH, gonadotropin-releasing hormone; LH, luteinizing hormone; LHRH, luteinizing hormone-releasing hormone; GIP, gonadotropin-independent puberty.

## Data Availability

The data that support the findings of this study are available from the corresponding author upon reasonable request.
